# The improvement effect of androgen priming on fertility in patients with diminished ovarian reserve and its impact on *in vitro* fertilization outcomes: a systematic review and meta-analysis

**DOI:** 10.3389/fendo.2026.1939223

**Published:** 2026-10-27

**Authors:** Xuanling Li, Chenlin Sui, Baofang Zhang, Yuquan Bai, Qian Zhao, Lianwei Xu

**Affiliations:** Department of Gynecology, Longhua Hospital, Shanghai University of Traditional Chinese Medicine, Shanghai, China

**Keywords:** androgen priming, diminished ovarian reserve, *in vitro* fertilization, meta-analysis, review

## Abstract

**Objective:**

Evaluate the effect of using androgen supplements (including transdermal testosterone gel and dehydroepiandrosterone (DHEA)) to priming androgens in the body and improve fertility in patients with diminished ovarian reserve (DOR).

**Methods:**

Electronic searches were conducted on seven databases, including Scopus, Web of Science, PubMed, Springer, VIP Information, Wanfang, and China National Knowledge Infrastructure (CNKI), to search for studies published as of July 5, 2026. Randomized controlled trails (RCTs) and non-randomized controlled trails of using androgen supplements (i.e. dehydroepiandrosterone (DHEA) and transdermal testosterone gel (TTG)) to intervene in improving fertility and IVF outcomes in patients with DOR were embodied. The study applied systematic review and meta-analysis methods to systematically summarize the main outcome measures, including FSH, AMH, AFC, retrieved oocytes, and clinical pregnancy rate. In addition, indicators such as the number of fertilized embryos, top-quality day-3 embryos, live birth rate and miscarriage rate were also evaluated.

**Results:**

The meta-analysis included 14 studies with 2132 participants. Analyses of RCTs and non-randomized controlled trials were conducted separately. A comprehensive analysis of existing evidence indicated that androgen supplementation, including DHEA and TTG, can improve count of retrieved oocytes [RCTs (MD = 0.79, 95% CI [0.50,1.09], *p*<0.00001)]. A retrospective analysis of non-randomized controlled trials showed that androgen supplementation can significantly increase the number of Top-quality day-3 embryos (MD 0.47, [95% CI: [0.28, 0.66], *p* < 0.00001), the count of fertilized embryos (MD 0.78, [95% CI: [0.49, 1.06], *p* < 0.00001), and clinical pregnancy rate (OR = 2.23, 95% CI [1.49,3.36], *p* = 0.0001). Besides, androgen supplementation showed Intervention with androgen supplements for 2 months was beneficial for retrieving more oocytes (*p* < 0.05) through the results in RCT. And also showed a more significant improvement in retrieved oocytes and metaphase II oocytes in DOR patients with an average age of over 40 years (*p* < 0.05). The subgroup analysis results indicated that the application of GnRH antagonists as IVF stimulation protocol was more beneficial in increasing the number of retrieved oocytes than the adopt of GnRH agonists.The sensitivity analysis concludes that our conclusion is robust.

**Conclusions:**

Androgen priming (including DHEA and TTG) may be beneficial for improving the fertility of DOR patients undergoing IVF, enhancing their ovarian response, mainly manifested as an increase in oocyte retrieval. However, whether this therapy can improve the clinical pregnancy rate and other outcome indicators of DOR women undergoing *in vitro* fertilization remains to be further determined by more randomized controlled trials. More high-quality and large sample RCTs are still needed in the future to clarify whether they have an auxiliary effect on improving the fertility outcomes of DOR.

**Systematic review registration:**

https://www.crd.york.ac.uk/PROSPERO/, identifier CRD420261438943.

## 1 Introduction

Infertility is a global reproductive health issue that affects 8% -12% of couples worldwide (1). One of the main causes of female infertility is diminished ovarian reserve (DOR), and according to statistics, 10-30% of female infertility is caused by DOR (2). DOR is a common female disease that can lead to decreased fertility and menstrual disorders (3), and can cause the ovaries to lose their normal reproductive potential, impair fertility, and result in a decrease in the number of oocytes (2, 4). Untreated patients often experience infertility, ovarian insufficiency, premature ovarian failure, and early onset or exacerbation of perimenopausal symptoms caused by estrogen fluctuations or deficiencies (5). The incidence rate of DOR showed a significant upward trend (6). According to the nationwide survey results in China from 2019 to 2020, the detection rate of ovarian reserve dysfunction in women of childbearing age (20–49 years old) is close to 30% (7).DOR is undoubtedly one of the common challenges currently faced in the field of assisted reproduction (8).

More and more evidence suggested that androgens play a crucial role in female reproductive physiology (9, 10). The expression of androgen receptor (AR) occurs at all levels of the hypothalamic pituitary gonadal axis, as well as other female reproductive organs or tissues such as the uterus, placenta, and immune system (11). AR mediated signaling mediates the regulation of important reproductive processes such as follicle development, embryo implantation, placental formation, and pregnancy maintenance by androgens. Animal experiments had shown that female mice lacking androgen receptors exhibited sub fertility and underdeveloped hair follicles (12), and moderate androgen supplementation could promote the development of preantral follicles in mice (13). Although long-term studies had shown that high levels of androgens can have multiple negative effects on fertility, in addition to inducing PCOS, hyperandrogenism can also interrupt the follicular maturation process, weaken the receptivity of the endometrium to the embryo, and exacerbate infertility and pregnancy loss (11, 14). But existing research also confirmed that maternal androgens are essential for successful pregnancy, and normal fertility requires a minimum level of androgens (13, 15, 16). Androgen deficiency may also lead to reproductive complications, and optimal androgen signaling is a fundamental requirement for female reproductive health. Insufficient androgens can disrupt key regulatory mechanisms (11, 17).

According to reports, circulating androgen levels decrease with age, especially in the early stages of reproduction (18). The process of reproductive aging involves a gradual decrease in the quantity and quality of oocytes, followed by an age-related decline in reproductive potentia (19). In light of these findings, IVF centers have begun to administer androgen pretreatment to patients with diminished ovarian reserve, with the aim of improving their reproductive outcomes (20).A previous survey showed that over 40% of doctors in Europe and Australia are prescribing off label androgens to patients with DOR (20). Systemic administration of androgens has been proposed as one of the adjuvant methods aimed at improving follicle recruitment and thus enhancing ovarian response to COS (21, 22). However, there is still controversy surrounding this therapy in clinical practice.

## 2 Objective(s)

This study aims to systematically evaluate the effects of androgen supplements (including DHEA and TTG) on fertility and IVF outcomes in DOR patients, and further explore whether appropriate androgen pretreatment can improve fertility outcomes in DOR patients, thereby providing scientific evidence for the importance of androgens in normal follicular development and female fertility.

## 3 Methods

This study followed the PRISMA guidelines (23) in methodology and has completed registration of the research protocol on the PROSPERO platform (CRD420261438943).

### 3.1 Eligibility criteria, information sources, search strategy

As of July 5, 2026, a total of 7 databases had been searched for all relevant research records since their establishment. Firstly, four English databases including Scopus, Web of Science, PubMed, and Springer were searched, followed by three Chinese databases including VIP Information, Wanfang, and China National Knowledge Infrastructure (CNKI). The search strategy consists of two parts: clinical diseases (diminished ovarian reserve, poor ovarian reserve) and intervention measures (testosterone, DHEA). Besides, we conducted an in-depth search and evaluation of relevant research references to obtain potential available studies.

### 3.2 Study selection

Studies that conform to the following criteria will be integrated: (1) those that meet the diagnostic criteria for DOR, namely 10 ≤ FSH ≤ 25 or AFC<7 or AMH<1.1, at least meet one or more of the above parameters (24, 25); (2) The DOR patients in the study received androgen priming therapy, including testosterone and DHEA; (3) This study reported the results of androgen priming therapy, including sex hormone levels or related IVF clinical parameters, providing at least sufficient data; (4) All clinical controlled trials evaluating the effectiveness of androgen priming (including testosterone and DHEA), including RCTs and non randomized controlled trials will be included, regardless of whether the control group uses a placebo. Regardless of the dosage of drugs used in the study or the duration of intervention, it can be included; (5) Research results published in Chinese or English.

In the selection process, excluded studies met the following criteria: (1) comorbidities with other diseases such as endometrial abnormalities, intrauterine adhesions, uterine malformations, and reproductive system tumors; (2) Research belongs to abstract, meta-analysis, reviews, duplicate publications, conference papers, and animal studies; (3) These studies have not yet been published in English or Chinese; (4) The study is a single arm anterior posterior control study. Due to its lack of parallel controls, it cannot be compared with RCTs or non randomized controlled trials.

### 3.3 Data extraction and assessment of risk of bias

Independently extract outcome indicators related to studies that meet the above qualification criteria using a pre designed table. The main research indicators included FSH, AMH, AFC, retrieved oocytes, and clinical pregnancy rate, while the secondary research indicators included Top-quality day-3 embryos, Metaphase II oocytes, Fertilized embryos, Live birth rate and miscarriage rate. Extract basic research information, including author surname, publication time, sample size, sample age, BMI, Types of androgen supplements, stimulation protocol, intervention doses, and intervention times, and record and summarize relevant outcome indicators included in the study. The quality of the included study (ROBINS-I and ROB2) was evaluated by two reviewers (X.L.L. and C.L.S.) using a two person double record method. Any disagreements that arise during the data organization process will be resolved through consultation with the third observer (L.W.X.).

### 3.4 Data synthesis and statistical analysis

Data management and statistical analysis were completed using EndNote 20.2, Stata/MP 17.0, and Review Manager 5.3, respectively. Continuous variables will be summarized using standardized mean difference (SMD) or mean difference (MD) and their 95% confidence intervals (CI). When the units of the results were consistent, MD was preferred, and SMD was adopted when the research units are different. The binary variables will be summarized using Relative Risk (RR) or odds ratio (OR) and their 95% CI. I^2^ statistical data was applied to evaluate heterogeneity between studies. When I^2^ ≤ 50%, a fixed effects model should be adopted. On the contrary, it was recommended to use a random effects model. Bilateral *p* ≤ 0.05 indicates statistically significant differences. Sensitivity analysis was applied to evaluate the impact of each study on the combined effect size, to test the robustness of meta-analysis conclusions, and to explore potential sources of heterogeneity and bias risk. Subgroup analysis was applied to explore the sources of heterogeneity. The detection of publication bias was implemented using a funnel plot. When the number of included studies reaches 10 or more, Begg’s test and Egger’s test were further supplemented for assessment.

## 4 Results

### 4.1 Study selection

According to the preliminary database search results, a total of 413 relevant literature records on the treatment of DOR with androgen priming (including DHEA and TTG) were retrieved. Out of 413 articles, 197 were duplicated and 193 were deleted due to not meeting the inclusion criteria. Subsequently, after carefully reading the included studies, we removed the other 9 studies as they lacked research data or did not belong to case-control studies. Finally, 14 case-control studies published between 2007 and 2026 were included. The selection process was shown in **Figure 1**.

**Figure 1:**
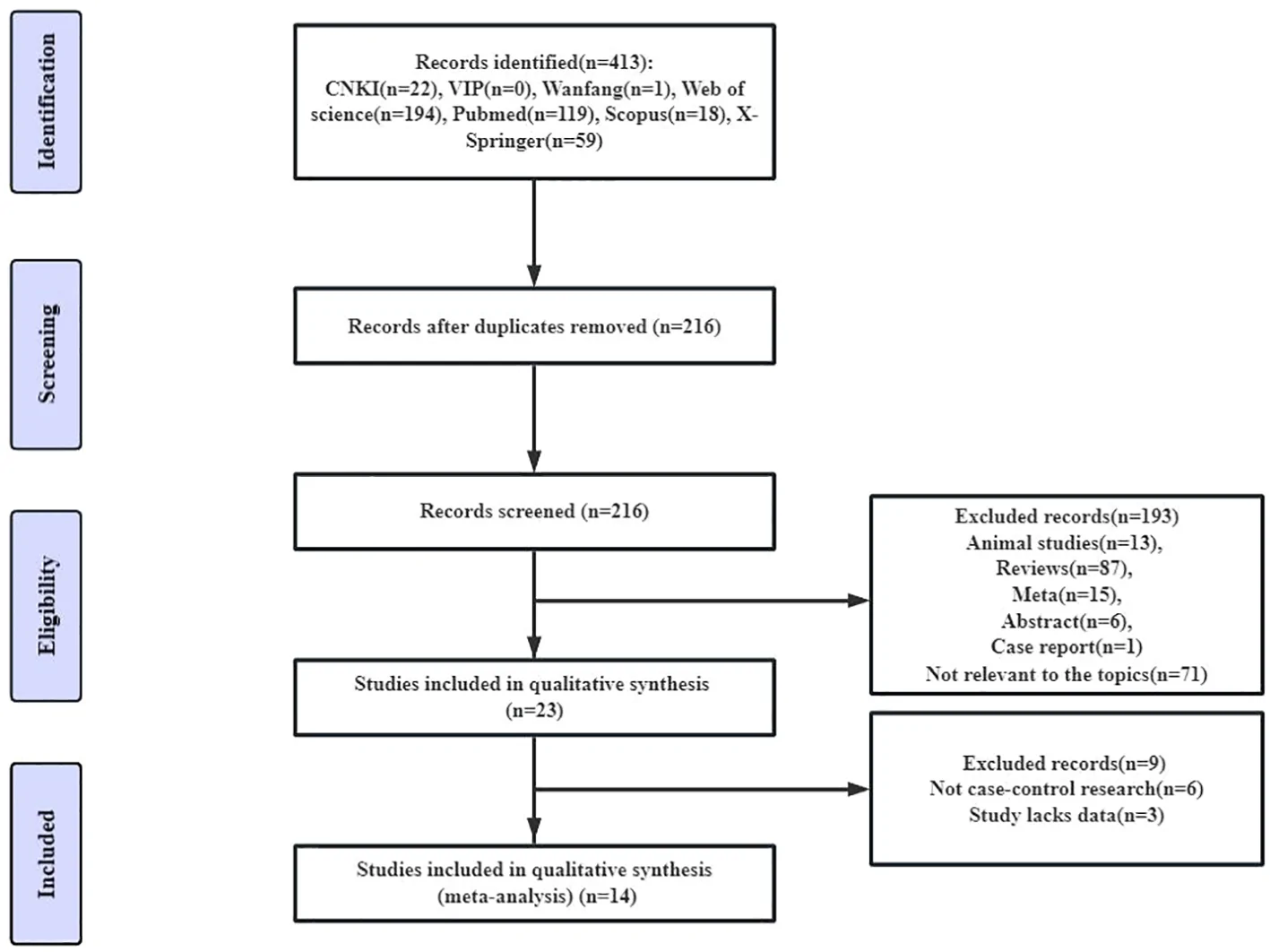
PRISMA literature screening process diagram.

### 4.2 Study characteristics

**Table 1** summarized the research characteristics of all included clinical trials, and this meta-analysis comprehensively evaluated a total of 14 studies. These studies involved a total of 2132 DOR patients, with sample sizes ranging from 18 to 486. Among these 14 trials, 9 were RCTs and 5 were non randomized controlled trials. And 4 were treated for 2 months (26–29), 7 for 3 months (30–36), 1 for 9 weeks (37), and 1 study for 4 months (38). Eleven studies investigated the efficacy of DHEA, and three studies investigated the efficacy of TTG. In addition, 5 studies chose GnRH agonists as the stimulation protocol, 6 studies chose GnRH antagonists as the stimulation protocol, and 1 study adopted mild controlled ovarian stimulation as the stimulation protocol.

**Table 1:** The principal features of the included studies.

Author	Year	Sample size(n)	stimulation protocol	Intervention	Dose	Intervention duration	Age	Body mass index (kg/m^2^)	Infertility duration (years)
RCT									
M. Kara (30)	2014	104	GnRH-agonist	DHEA	25 mg three times a day	12 weeks	30.97 ± 5.76	/	5.31 ± 1.23
		104	GnRH-agonist	control	/		31.15 ± 5.58	/	6.09 ± 1.02
Tartagni (26)	2015	53	GnRH-agonist	DHEA	75 mg daily	8 weeks	37.1 ± 2.2	22.1 ± 1.6	/
		56	GnRH-agonist	Placebo	75 mg daily		37.4 ± 2.9	22.5 ± 1.4	/
Wang (32)	2016	24	/	DHEA	25 mg three times a day	3 months	31.6 ± 4.0	22.7 ± 3.3	4.8 ± 2.6
		19	/	control	/		31.6 ± 4.8	23.3 ± 2.8	4.3 ± 3.5
Fu (33)	2017	48	mild controlled ovarian stimulation	DHEA	25 mg three times a day	12 weeks	37.4 ± 3.6	21.60 ± 2.75	4.39 ± 2.07
		43	mild controlled ovarian stimulation	control	/		36.8 ± 4.3	22.13 ± 2.45	4.58 ± 1.51
Lin (28)	2017	28	GnRH antagonists	DHEA	90 mg daily	2 months	40.2 ± 1.7	21.5 ± 2.0	5.6 ± 4.2
		30	GnRH antagonists	Control	/		40.7 ± 2.0	21.2 ± 4.2	6.5 ± 5.5
Agarwal (34)	2017	20	/	DHEA	25 mg three times a day	12 weeks	33.10 ± 4.29	25.01 ± 3.31	/
		20	/	Placebo	25 mg three times a day		32.30 ± 4.07	25.04 ± 3.77	/
Nayar (39)	2021	25	GnRH antagonists	transdermal testosterone gel	12.5 mg daily	from day 6th of previous cycle to day 2nd of stimulation cycle	/	/	/
		25	GnRH antagonists	lubricant gel	12.5 mg daily		/	/	/
Bruno (29)	2025	9	GnRH antagonists	transdermal testosterone gel	1% 25mg daily	8 weeks	36.7 ± 3.8	22.5 ± 3.0	3.5 ± 1.8
		9	GnRH antagonists	control	/		37.6 ± 2.9	22.8 ± 2.9	5.1 ± 4.2
Polyzos (37)	2026	134	GnRH-agonist	transdermal testosterone gel	1% 5.5 mg daily	9 weeks	38.0 ± 3.5	24.9 ± 4.6	11.5 ± 2.8
		154	GnRH-agonist	Placebo	5.5 mg daily		38.1 ± 3.2	24.2 ± 4.0	11.7 ± 2.2
Non randomized studies									
Barad (38)	2007	89	GnRH-agonist	DHEA	75 mg daily	4 months	41.6 ± 0.4	/	/
		101	GnRH-agonist	control	/		40.0 ± 0.4	/	/
Liu (31)	2016	243	GnRH-agonist	DHEA	25 mg three times a day	3 months	39.05 ± 1.59	24.30 ± 2.99	7.76 ± 4.95
		243	GnRH-agonist	control	/		38.86 ± 1.66	24.16 ± 3.05	7.37 ± 4.85
Hu (27)	2017	53	GnRH antagonists	DHEA	25 mg three times a day	8 weeks	33.28 ± 3.13	22.32 ± 2.44	3.81 ± 2.61
		50	GnRH antagonists	control	/		34.16 ± 3.27	23.24 ± 4.41	3.86 ± 2.56
Chern (36)	2018	67	GnRH antagonists	DHEA	90 mg daily	3 months	39.1 ± 3.3	21.9 ± 2.9	5.6 ± 4.2
		84	GnRH antagonists	Control	/		39.8 ± 3.7	21.8 ± 3.8	5.3 ± 4.5
Chen (35)	2019	159	GnRH antagonists	DHEA	30 mg three times a day	3 months	39.8 ± 2.5	22.3 ± 3.1	5.6 ± 4.6
		138	GnRH antagonists	Control	/		40.2 ± 2.9	23.0 ± 3.7	4.9 ± 4.3

### 4.3 Risk of bias of included studies

For non-randomized controlled trails, the Cochrane ROBINS-I tool was applied for quality assessment (**Figure 2**). For RCTs, Cochrane RoB 2.0 tool was adopted for quality assessment (**Figure 3**). Among all included studies, 5 studies did not use random allocation, and 11 trials did not implement blinding throughout the entire study process, thus being considered to have a “high”, “serious” “ moderate” or “some concern” risk of bias. However, due to insufficient explanation of the specific details of the outcome evaluation process in most studies, the actual differentiation of scores (i.e. the basis for determining whether a study is “high” or “low”) may be influenced by bias. In these included studies, we did not find any details of selective reporting. Therefore, we believed that the main risk of bias lies in the blindness and randomness of this meta-analysis.

**Figure 2:**
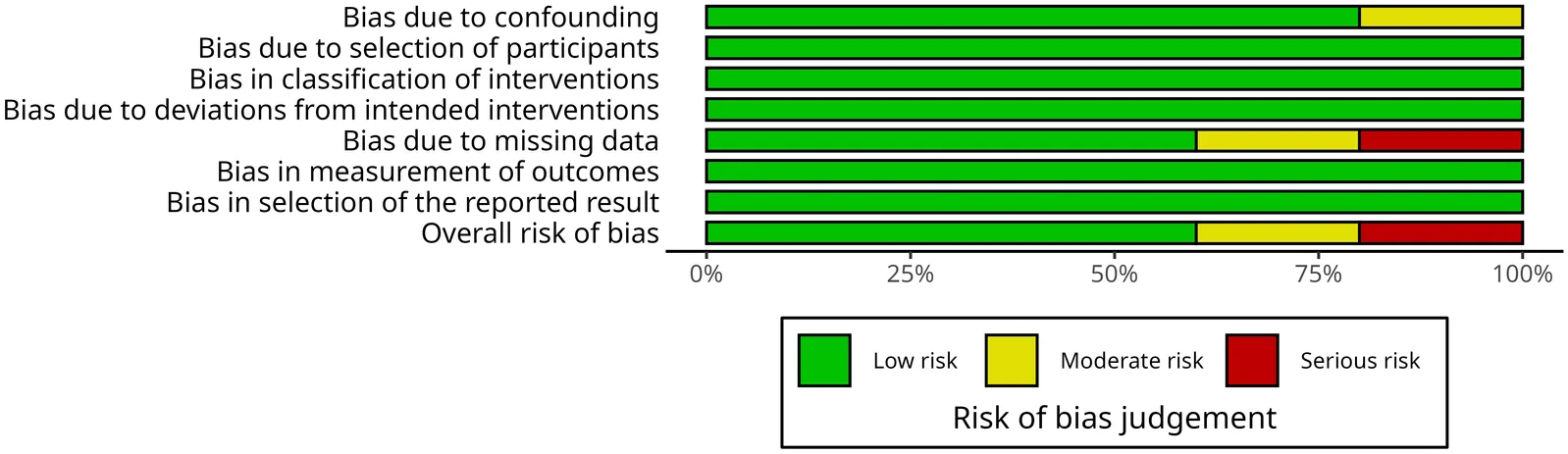
Risk of bias summary in five non-randomized controlled trails (ROBINS-I).

**Figure 3:**
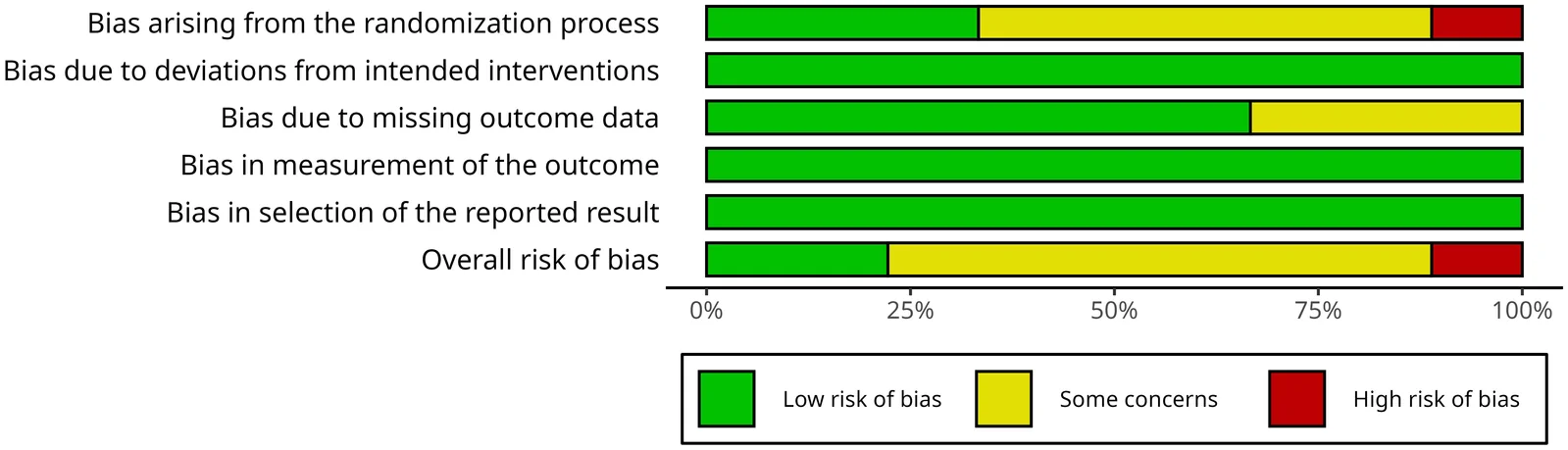
Risk of bias summary in nine RCTs (ROB2).

### 4.4 Synthesis of results

#### 4.4.1 Main research indicators

To avoid selection bias and confusion about the true size of the effects, we conducted statistical analysis on the main outcome measures of both randomized controlled trials and non-randomized controlled trails seperatly. After intervention with androgen priming, RCT analysis results showed an increase in AMH levels (SMD = 0.08, 95% CI [-0.27, 0.42], *p* = 0.66) (**Figure 4B**), a decrease in FSH levels (SMD=-0.08, 95% CI [-0.40, 0.24], *p* = 0.63) (**Figure 4C**), and an enhancement in AFC levels (MD = 0.23, 95% CI [-0.21, 0.67], *p* = 0.31) (**Figure 4A**) of DOR patients. Only one non randomized controlled study (27) reported AMH, AFC, and FSH. This study included 103 DOR patients and compared DHEA with a control group. After 8 weeks of intervention, the AMH of the intervention group and the control group were [0.96 ± 0.16] vs [0.89 ± 0.20], respectively (MD [0.38], 95% CI [-0.01,0.77], P = 0.05); The AFC values were [5.34 ± 1.68] vs [4.66 ± 1.44] (MD [0.68], 95% CI [0.08, 1.28], P = 0.03); FSH levels were [9.51 ± 1.78] vs [10.16 ± 2.48] (MD [-0.30], 95% CI [-0.69,0.09], P = 0.13). Due to the non randomized design of the study, it cannot be combined with randomized controlled trials (RCTs) for analysis. Therefore, these results were only presented as descriptive reports and no meta-analysis had been conducted. Although the statistical results of RCTs had a p-value greater than 0.05, we believed that this may be due to the fact that the outcome data in the studies we included only involved 2–3 studies, resulting in limited data. In the statistical results of IVF outcome indicators, 12 studies recorded retrieved oocytes, and the analysis results showed that intervention with androgen priming before IVF could improve retrieved oocytes in DOR patients [RCTs (MD = 0.79, 95% CI [0.50,1.09], *p*<0.00001) (**Figure 4D**), non-randomized controlled trails (MD = 0.11, 95% CI [-1.63,1.86], *p* = 0.9)] (**Figure 4F**). In addition, 10 studies involved clinical pregnancy rates, including 6 RCTs and 4 non-randomized studies. The comprehensive results showed that the adopt of androgen pretreatment may be beneficial for increasing the clinical pregnancy rate of DOR patients [RCTs (OR = 1.23, 95% CI [0.87,1.73], *p* = 0.24) (**Figure 4E**), non-randomized controlled trails (OR = 2.23, 95% CI [1.49,3.36], *p* = 0.0001)] (**Figure 4G**).

**Figure 4:**
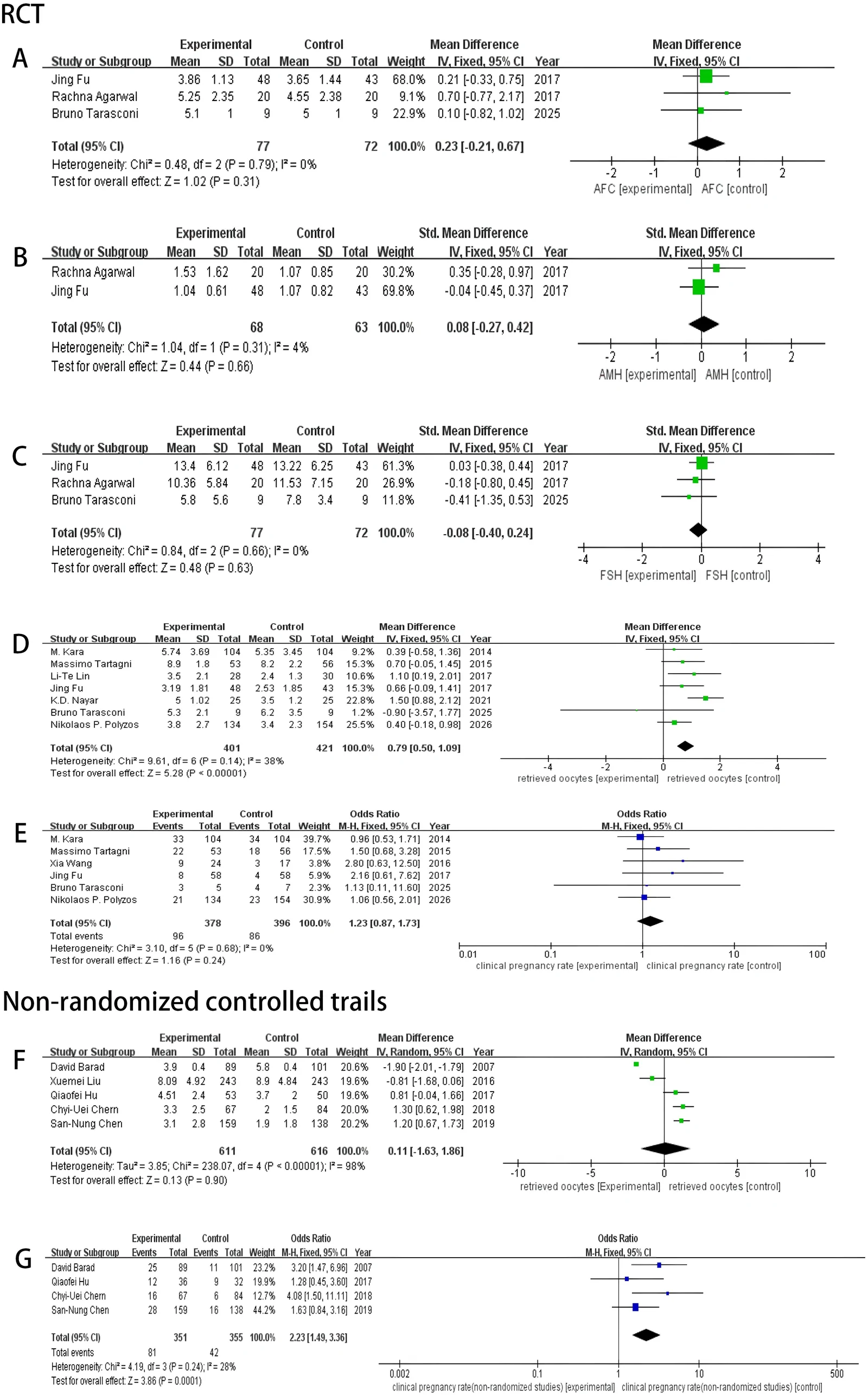
Forest plot: the main outcome measure of androgen priming ameliorating DOR. RCT: **(A)** AFC; **(B)** AMH; **(C)** FSH; **(D)** retrieved oocytes; **(E)** clinical pregnancy rate; Non-randomized controlled trails: **(F)** retrieved oocytes; **(G)** clinical pregnancy rate.

#### 4.4.2 Secondary research indicators

So far, clinical evaluation of embryo quality has often been based on examining the number, degree of fragmentation, and uniformity of blastomeres. According to Scott’s standards, embryos that grow to the third day and have six or more cells are considered high-quality embryos (40). In 7 studies the number of metaphase II oocytes was recorded, in 4 studies the number of top-quality day-3 embryos obtained by DOR patients was recorded, in 5 studies the live birth rate was recorded, and in 5 studies the miscarriage rate was recorded. We conducted statistical analyses on RCT and non randomized studies separately. Among them, the comprehensive analysis results of non-randomized controlled trials indicated that androgen priming can increase the number of top-quality day-3 embryos (MD = 0.47, 95% CI [0.28,0.66], *p*<0.00001) and fertilized embryos (MD = 0.78, 95% CI [0.49,1.06], *p*<0.00001).

It is worth noting that RCT analysis showed that androgen priming can increase the number of metaphase II oocytes [RCT (MD 0.12, [95% CI: -0.44,0.68], *p* = 0.68)] compared to no intervention. Moreover, pretreatment of androgen could increase live birth rate of DOR patients [RCT (OR 1.24, [95% C1:0.74, 2.08], *p* = 0.41) and lower the miscarriage rate [RCT (OR 0.39, [95% C1:0.04, 3.42], *p* = 0.40)]. Although the difference did not reach the predetermined statistical significance level (*p*>0.05), this may be due to insufficient testing power caused by limited sample size. The comprehensive results suggested that androgen priming has a potential clinical benefit tendency, showing favorable numerical trends. **Table 2** listed all the results mentioned above.

**Table 2:** Analysis of secondary research indicators results.

Clinical results	Studies (n)	Participants (n)	OR/SMD/MD 95% CI	*p*	I^2^(%)	Model
RCT						
Top-quality day-3 embryos	2	346	0.57 [-0.02, 1.15]	0.06	80	Random
Metaphase II oocytes	6	772	0.12[-0.44,0.68]	0.68	78	Random
Live birth rate	3	415	1.24 [0.74, 2.08]	0.41	49	Fixed
Miscarriage rate	4	104	0.39 [0.04, 3.42]	0.40	63	Random
Non-randomized controlled trails						
Fertilized embryos	3	551	0.78 [0.49, 1.06]	<0.00001	0	Fixed
Top-quality day-3 embryos	2	448	0.47 [0.28, 0.66]	<0.00001	0	Fixed
Live birth rate	2	448	1.72 [0.93, 3.18]	0.08	38	Fixed

#### 4.4.3 Subgroup analysis

Considering the significant heterogeneity (I^2^ ≥ 50%) in the comprehensive results of outcome indicators such as Metaphase II oocytes and high-quality 3rd day embryos, subgroup analysis was performed. The comprehensive results of RCT revealed that in the subgroup analysis of DHEA and TTG, the adopt of DHEA before the start of the IVF cycle could effectively increase the count of retrieved oocytes (*p* = 0.0007) and Metaphase II oocytes (*p* = 0.009), and significantly reduce the miscarriage rate (*p* = 0.01). And the comprehensive analysis of non-randomized controlled trials showed that DHEA pretreatment can effectively increase the number of top-quality day-3 embryos(*p*<0.00001) and significantly improve the clinical pregnancy rate (*p* = 0.0001). It is worth noting that both the adopt of TTG or DHEA intervention before the start of the IVF cycle can increase the count of retrieved oocytes. Although the count of retrieved oocytes after the intervention of TTG is slightly better than that of DHEA. However, in the included randomized controlled trial studies, DHEA showed statistically significant improvements in certain subgroups compared to the control group, while TTG did not show statistically significant improvements. Due to the lack of direct comparative studies between DHEA and TTG, and TTG related evidence only comes from three randomized controlled trials, it is currently impossible to infer that DHEA is superior to TTG. Which of the two is more effective still needs to be verified through direct comparison through randomized controlled trials or relevant network meta-analyses.

In addition, in terms of intervention time, using androgen activation therapy 2 months before the start of the IVF cycle can obtain more retrieved oocytes (*p* = 0.008) through the analysis of RCTs. It is worth noting that David Barad et al.’s (38) study used androgen activation therapy to intervene for 4 months, although the clinical pregnancy rate increased and the miscarriage rate decreased, the number of retrieved oocytes was less than that of the control group. Based on our comprehensive analysis of the subgroup analysis results of randomized controlled trials, we found that with the prolongation of intervention time, the clinical pregnancy rate showed a decreasing trend, but the difference was not statistically significant. Subsequently, we conducted subgroup analysis for different age groups. The results indicated that androgen pretreatment is more effective in women with an average age over 40 years old than in women under 40 years old. Among these DOR patients, a comprehensive analysis of RCTs observed an increase in the number of retrieved oocytes (*p* = 0.003) and metaphase II oocytes (*p* = 0.005) after intervention in DOR patients with an average age over 40 years old. Subgroup analysis of non randomized controlled trials also showed that after intervention, the clinical pregnancy rate of women with an average age over 40 years old significantly increased (*p* < 0.0001). It is worth noting that through subgroup analysis, we found that choosing GnRH antagonists after androgen pretreatment has a more significant effect on the number of retrieved oocytes (*p* = 0.002) after IVF compared to GnRH agonists. **Table 3** displayed the subgroup analysis results for different intervention measures, intervention times, intervention ages, and stimulation protocols. To avoid the increased risk of type I (false positive) errors caused by repeated statistical testing, we supplemented the Benjamini Hochberg False Discovery Rate (BH-FDR) correction for intra subgroup comparisons and reported uncorrected P-values and corrected q-values separately.

**Table 3:** Subgroup analysis.

Clinical results	Studies (n)	Participants (n)	OR/SMD/MD 95% CI	*p*	q (BH-FDR)	I^2^(%)	Model
DHEA (RCT)							
retrieved oocytes	4	466	0.72[0.30,1.13]	0.0007	0.0028	0	Random
metaphase II oocytes	4	466	0.40[0.10,0.70]	0.009	0.0133	37	Fixed
Clinical pregnancy rate	4	474	1.31[0.86,1.98]	0.2	0.2	0	Fixed
Miscarriage rate	2	52	0.06[0.01,0.54]	0.01	0.0133	0	Fixed
DHEA (Non-randomized controlled trails)							
retrieved oocytes	5	1227	0.11[-1.63,1.85]	0.90	0.9	98	Random
top-quality day-3 embryos	2	448	0.47[0.28,0.66]	<0.00001	<0.00004	0	Fixed
Clinical pregnancy rate	4	706	2.23[1.49,3.36]	0.0001	0.0002	28	Fixed
live birth rate	2	448	1.72[0.93,3.18]	0.08	0.1067	38	Fixed
Testosterone (RCT)							
retrieved oocytes	3	356	0.73[-0.31,1.77]	0.17	0.625	76	Random
metaphase II oocytes	2	306	0.22[-0.29,0.74]	0.4	0.6625	59	Fixed
Clinical pregnancy rate	2	300	1.06[0.57,1.98]	0.85	0.85	0	Fixed
live birth rate	2	306	0.81[0.41,1.59]	0.53	0.6625	0	Fixed
Miscarriage rate	2	52	2.22[0.55,8.75]	0.25	0.625	0	Fixed
2 months (RCT)							
retrieved oocytes	3	185	0.78[0.20,1.36]	0.008	0.025	2	Random
metaphase II oocytes	3	185	0.48[0.10,0.86]	0.01	0.025	69	Fixed
Clinical pregnancy rate	2	121	1.46[0.69,3.06]	0.32	0.32	0	Fixed
live birth rate	2	127	2.06[0.97,4.39]	0.06	0.075	0	Fixed
Miscarriage rate	2	48	0.16[0.02,1.07]	0.06	0.075	43	Fixed
3 months (RCT)							
AFC	2	131	0.27[-0.24,0.77]	0.3	0.4875	0	Fixed
FSH	2	131	-0.03[-0.38,0.31]	0.85	0.85	0	Fixed
retrieved oocytes	2	299	0.56[-0.04,1.15]	0.07	0.35	0	Random
metaphase II oocytes	2	299	0.21[-0.27,0.69]	0.39	0.4875	0	Fixed
Clinical pregnancy rate	3	365	1.24[0.76,2.03]	0.39	0.4875	21	Fixed
3 months (Non-randomized controlled trails)							
retrieved oocytes	3	934	0.60[-0.56,1.76]	0.31	0.31	89	Random
Clinical pregnancy rate	2	448	2.18[1.26,3.76]	0.005	0.015	55	Fixed
live birth rate	2	448	1.72[0.93,3.18]	0.08	0.12	38	Fixed
> 2 months (RCT)							
AMH	2	131	0.08[-0.27,0.42]	0.66	0.78	4	Fixed
retrieved oocytes	3	587	0.48[0.06,0.89]	0.02	0.1	0	Random
metaphase II oocytes	3	587	0.25[-0.10,0.61]	0.16	0.4	0	Fixed
Clinical pregnancy rate	4	653	1.17[0.79,1.73]	0.43	0.7167	0	Fixed
Miscarriage rate	2	56	1.19[0.35,4.07]	0.78	0.78	77	Fixed
> 2 months (Non-randomized controlled trails)							
retrieved oocytes	4	1124	-0.06[-2.03,1.91]	0.95	0.95	99	Random
top-quality day-3 embryos	2	448	0.47[0.28,0.66]	<0.00001	<0.00004	0	Fixed
Clinical pregnancy rate	3	638	2.47[1.58,3.86]	<0.0001	<0.0002	31	Fixed
live birth rate	2	448	1.72[0.93,3.18]	0.08	0.1067	38	Fixed
<40 years old (RCT)							
retrieved oocytes	3	335	0.51[-0.07,1.09]	0.08	0.1333	0	Random
metaphase II oocytes	3	335	0.19[-0.19,0.56]	0.33	0.360	24	Fixed
Clinical pregnancy rate	4	370	1.22[0.79,1.89]	0.36	0.360	0	Fixed
live birth rate	2	127	2.06[0.96,4.42]	0.06	0.1333	0	Fixed
Miscarriage rate	3	60	0.13[0.03,0.70]	0.02	0.100	4	Fixed
>40 years old (RCT)							
retrieved oocytes	3	437	0.62[0.21,1.03]	0.003	0.0075	0	Random
metaphase II oocytes	3	437	0.51[0.15,0.88]	0.005	0.0075	40	Fixed
Clinical pregnancy rate	2	404	1.24[0.70,2.18]	0.47	0.470	0	Fixed
>40 years old (Non-randomized controlled trails)							
retrieved oocytes	4	1124	-0.06[-2.03,1.91]	0.95	0.950	99	Random
Clinical pregnancy rate	3	638	2.47[1.58,3.86]	<0.0001	<0.0003	31	Fixed
live birth rate	2	448	1.72[0.93,3.18]	0.08	0.120	38	Fixed
Stimulation Plan - GnRH-agonist (RCT)							
retrieved oocytes	3	605	0.49[0.07,0.91]	0.02	0.06	0	Random
metaphase II oocytes	3	605	0.25[-0.05,0.56]	0.11	0.165	0	Fixed
Clinical pregnancy rate	3	705	1.10[0.75,1.6]	0.62	0.62	0	Fixed
Stimulation Plan - GnRH-agonist (Non-randomized controlled trails)							
retrieved oocytes	2	676	-1.44[-2.5,-0.39]	0.007	0.007	83	Random
Stimulation Plan - GnRH antagonists (RCT)							
retrieved oocytes	3	126	1.17[0.43,1.92]	0.002	0.004	38	Random
metaphase II oocytes	2	76	0.90[0.20,1.60]	0.01	0.01	77	Fixed
Stimulation Plan - GnRH antagonists (Non-randomized controlled trails)							
retrieved oocytes	3	551	1.11[0.58,1.64]	<0.0001	<0.0002	0	Random
Clinical pregnancy rate	3	516	1.94[1.20,3.14]	0.007	0.007	33	Fixed

### 4.5 Publication bias

Due to *p*>0.05, funnel plots were plotted for the main outcome measures AMH (*p* = 0.66), FSH (*p* = 0.63), AFC (*p* = 0.31), retrieved oocytes (non randomized studies, *p* = 0.9) and clinical pregnancy rate (RCTs, *p* = 0.24) in this meta-analysis, and no prominent asymmetry was examined in the funnel plots (**Figures 5A–E, I, J**). In addition, we evaluated the overall impact of individual studies by measuring the count of retrieved oocytes, mid-term II oocytes, and clinical pregnancy rate through sensitivity analysis and assessed the robustness of the results (**Figures 5F–H, K, L**). No significant statistical differences were discovered in the results, indicating that the meta results are stable.

**Figure 5:**
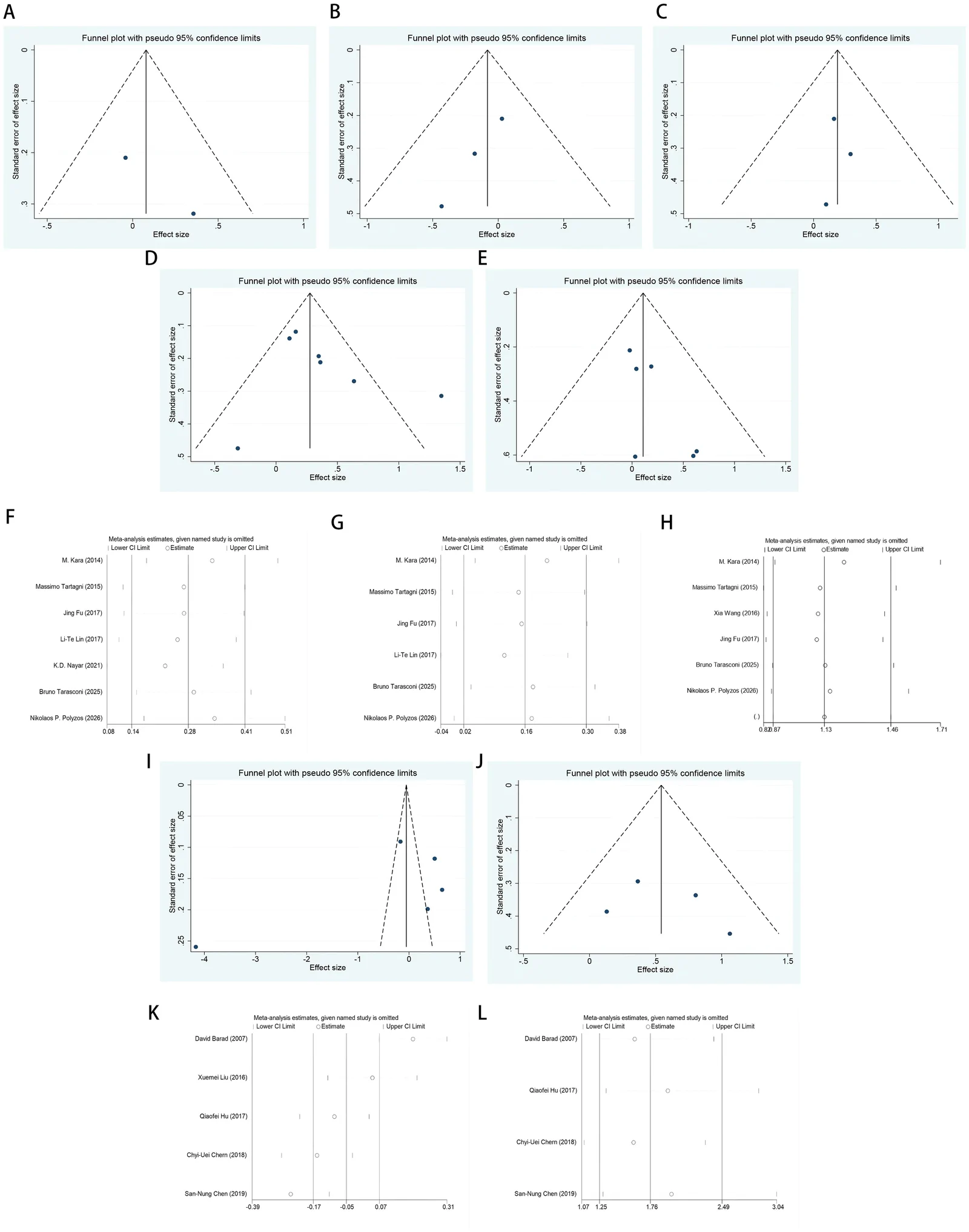
Publish bias analysis chart. RCT: Funnel chart **(A)** AMH; **(B)** FSH; **(C)** AFC; **(D)** retrieved oocytes; **(E)** clinical pregnancy rate; Sensitivity analysis **(F)** retrieved oocytes; **(G)** metaphase II oocytes; **(H)** clinical pregnancy rate; Non-randomized controlled trails: Funnel chart **(I)** retrieved oocytes; **(J)** clinical pregnancy rate; Sensitivity analysis **(K)** retrieved oocytes; **(L)** clinical pregnancy rate.

## 5 Discussion

Our meta-analysis aims to evaluate whether androgen priming can improve the fertility of DOR women and improve the reproductive outcomes of IVF in DOR women. We included a total of 14 clinical controlled studies involving 2132 DOR patients. Through meta-analysis, we evaluated the levels of FSH, AMH, AFC, and assessed the number of retrieved oocytes, top-quality day-3 embryos, fertilized embryos, clinical pregnancy rate, live birth rate, and miscarriage rate of DOR patients after IVF were evaluated. The meta-analysis results of randomized controlled trials (RCTs) and non randomized controlled trials indicated that androgen priming is beneficial in increasing the count of retrieved oocytes (*p* < 0.05), and may have potential therapeutic effects on improving clinical pregnancy rates after IVF. The subgroup analysis results revealed that the use of DHEA before the start of IVF cycles can effectively increase the number of retrieved oocytes and metaphase II oocytes, and can reduce miscarriage rates. However, as we only retrieved three studies on TTG intervention in DOR, more and larger randomized controlled trials are still needed to further explore and elucidate the specific effects of androgen pretreatment and to further clarify which one is more effective, DHEA or TTG.

DOR is a core pathological condition that leads to a decline in female fertility, manifested as a decrease in the number of follicles and a decrease in the quality of ovarian oocytes, significantly affecting the outcomes of natural pregnancy and assisted reproductive technologies (41). Numerous animal studies had shown that insufficient androgen activity in women can lead to low fertility, which is very similar to the clinical manifestations of female DOR (42). In the female endocrine and reproductive network, androgens (such as DHEA, DHEA-S, androstenedione (A4), testosterone (T), and dihydrotestosterone (DHT) play an indispensable regulatory role and are important hormone components that maintain the normal function of this physiological systems (43). DHEA, DHEA-S, and A4 must be biotransformation into T or DHT in order to exert physiological effects. Subsequently, T or DHT binds to the AR, activating downstream signaling pathways to induce various biological effects, including follicular development (44). It is worth noting that FSH stimulates the expression of P450 aromatase, which is a key enzyme in the biosynthesis of 17β- estradiol by androgens (16, 45). When androgens are converted into estradiol by granulosa cell aromatase, they can bind with AR to exert androgenic effects, thereby affecting follicle growth, increasing steroidogenesis, promoting follicle recruitment, and increasing the number of primary and pre antral follicles (43, 46). Androgens regulate all stages of follicular maturation and are crucial for coordinating ovarian function and fertility. During early follicular development, androgens are involved in promoting the transition of follicles from quiescent pool to growth pool (47). In the pre-antral stage, androgens from the follicular membrane bind to AR in granulosa cells, inducing the expression of follicle stimulating hormone receptor (FSHR) and follicle growth (48). At the same time, androgens can increase FSHR protein levels, enhance follicle sensitivity to FSH, and accelerate progression towards antral follicles (42). In the late follicular stage, androgens promote the maturation of oocytes and participate in ovulation (10). Mouse studies had confirmed that threshold androgen doses could stimulate rapid follicular maturation (49). In addition, studies had found that the application of AR antagonists could impair the AR mediated ovulation process, leading to reduced ovulation (16). Overall, androgens can not only reduce follicular atresia and granulosa cell apoptosis, but also participate in stimulating granulosa cell proliferation and differentiation (11).

The traditional view is that androgens have a damaging effect on ovarian function, and their abnormal levels are often closely related to infertility (50). It is worth noting that with the continuous accumulation of various androgen receptor (AR) knockout mouse models, *in vitro*/*in vivo* experiments, and clinical data, it had been proven that appropriate androgen AR pathway activity is necessary for the normal growth and function of follicles (13, 51, 52). Androgens play a double-edged sword role in ovarian function. On the one hand, moderate levels of androgens are essential for follicular development. On the other hand, excessive levels of androgens can in turn inhibit the normal formation of antral follicles and lead to follicular development disorders, which is an important pathogenesis of PCOS (53). And low androgen levels may be associated with abnormal follicular growth, LFOR, and POI, which can have a negative impact on female fertility (45). Obviously, the key is to maintain precise balance.

In the light of data from the Centers for Disease Control and Prevention in the United States, DOR was the third most common indicator for IVF in 27% of IVF cycles in 2021 (37). Research had shown that pre-treatment with androgens before ovarian stimulation during IVF cycles can upregulate the expression of FSH receptors on granulosa cells (GC), thereby enhancing follicle response to exogenous FSH. On the other hand, LH and hCG drive the synthesis of androgens in follicles by binding to LH receptors on follicular membrane cells, providing a theoretical basis for ovarian androgen activation strategies (54). Exogenous testosterone and DHEA activation had been shown to increase the count of retrieved oocytes and live birth rate (55). DHEA is a weak androgen and an important precursor to sex steroids, which can be converted into androgens and estrogens depending on the hormonal milieu (56). According to reports, DHEA can increase oocyte/embryo yield and oocyte/embryo quality in women with DOR (57). Research had shown that DHEA may regulate hormone receptors in the ovaries and hormone secretion in the peripheral circulation to regulate ovarian reserve function (58). Another study by Ma et al. (59) found that DHEA treatment can reduce the severity of DOR and enhance ovarian reserve function by weakening AMPK-SIRT1 signaling and mitochondrial autophagy in rat ovaries. In addition, multiple studies had shown that DHEA can increase oocyte and embryo production, increase pregnancy rates, and reduce miscarriage rates (57, 60). A meta-analysis suggested that the apply of DHEA in POR patients can increase pregnancy rates, reduce miscarriage rates, and does not affect average oocyte retrieved rate (61). In addition, androgen therapy with testosterone gel had been proven to have potentially beneficial effects on ovarian response and pregnancy outcome (37). The transdermal absorption range of testosterone is about 9% to about 12% of the applied dose (62). This transdermal delivery system allows testosterone to enter the bloodstream smoothly within 24 hours after absorption, with a relatively constant release rate, thereby ensuring that serum T levels remain within a stable range (62). Research had shown that in the IVF/ICSI cycle, androgen pretreatment with TTG (transdermal testosterone gel) can effectively enhance the ovarian response to controlled ovarian stimulation (COS), and improve the clinical pregnancy rate, so as to maximize the fertility of patients with low ovarian response (63). Animal experiments had confirmed that testosterone treatment can initiate follicular growth, upregulate FSH receptor expression, enhance ovarian sensitivity to FSH, and may ultimately promote oocyte maturation by inhibiting cell apoptosis and follicular atresia (22).

According to our search results, this research is the first meta-analysis and systematic review of clinical case-control trails on androgen priming in DOR patients. In addition, unlike other similar studies, this study conducted subgroup analysis around different stimulation protocol, intervention measures, intervention duration, and intervention age groups. The summary of subgroup analysis for different intervention times shows that the effect of androgen pretreatment for 2 months is more significant. However, due to the limited number of studies included and the small sample size, it is not possible to determine the optimal intervention time based on this. A comprehensive analysis of RCTs observed that DOR patients with an average age over 40 years old had an increased number of retrieved oocytes (p=0.003) and mid-term II oocytes (p=0.005) after receiving androgen intervention, with better outcomes compared to women under 40 years old. Research had shown that low androgen levels are associated with DOR in all age groups (22, 64). Androgens mainly come from adrenal precursor substances, and DOR to some extent reflects the clinical dysfunction status of the adrenal reticular zone (65). As age increases, the secretion of androgen precursors by the adrenal gland rapidly decreases, which is more prominent in elderly women (64). Research suggested that young women are more effective in converting androgens than older women, and older women may benefit from more direct androgen administration (64). This is consistent with our analysis results. However, it should be noted that due to the limited sample size, our statistics have limitations. Besides, to avoid potential driving factors affecting the results of androgen priming, we conducted a subgroup analysis of the stimulation protocol for IVF, and the results prompted that compared with GnRH agonists, choosing GnRH antagonists after androgen priming had a more significant impact on the number of retrieved oocytes after IVF (p=0.002). Currently, GnRH antagonist regimens are commonly used for DOR due to their flexibility and safety (66). So far, clinical applications have demonstrated several advantages of this regimen for women, including shortened stimulation time, reduced total dose of gonadotropins required for ovarian stimulation, no risk of cyst formation, no hormone withdrawal symptoms, and reduced incidence of ovarian hyperstimulation symptoms (67). The latest study (68) confirmed that the antagonist regimen is associated with shorter stimulation and lower consumption of gonadotropins compared to the long-term GnRH agonist regimen, making it more cost-effective. The comprehensive conclusion indicated that the use of androgen priming before IVF has potential beneficial effects on the pregnancy outcomes of DOR patients.

It should be pointed out that our systematic reviews still have limitations. Firstly, different androgen supplements have different effects, sensitivity, and specificity; Secondly, there are differences in the research methods and inclusion criteria of clinical trials in the studies we included, which may also have a certain impact on the research results. The differences in intervention time and sample size among various studies may also lead to increased heterogeneity; Moreover, multiple studies included did not provide sufficient and detailed baseline data, especially incomplete reports on patients’ occupational background, education level, and other aspects. This deficiency seriously restricts us from conducting more refined subgroup analysis and hinders the feasibility of exploring potential sources of heterogeneity through meta regression. Moreover, due to the fact that the number of studies included in each outcome indicator is less than 10, Begg’s and Egger tests were not performed to evaluate publication bias. Although no obvious asymmetry was found in the funnel plot, its explanatory value is relatively limited due to the limited number of studies. We conducted separate meta-analyses of the included RCTs and non randomized controlled trials. Although this meta-analysis suggested that androgen pretreatment may have a potential beneficial trend in improving fertility in DOR patients, we can only provide weak recommendations due to limitations in the quality or quantity of evidence. That is to say, androgen pretreatment, including DHEA and TTG, may serve as adjuvant therapy to help activate androgens, thereby improving the fertility of DOR patients and enhancing the reproductive outcomes of IVF.

At present, there is still controversy over whether androgens are beneficial for normal follicular maturation and female fertility in humans (69). Testosterone gel preparation is considered as a popular testosterone replacement therapy in patients with hypogonadism. This gel preparation can restore the testosterone level to a moderate physiological level (14-17.5 nmol/L) within 24 hours, thereby helping to reduce the signs and symptoms of testosterone deficiency (70). Although compared with injections and patches, testosterone gel has good efficacy and safety, but the risk of secondary exposure is still a challenge (70). In addition, it should be pointed out that long-term use of dehydroepiandrosterone may lead to adverse reactions such as acne, stomach discomfort, dizziness, etc (71). In the studies included in our meta-analysis, 7 research did not show any adverse reactions, and 4 studies did not state whether adverse reactions occurred. After using DHEA intervention, one study reported mild transient acne, oily skin, and mild hair loss in a small number of patients, and another study reported one patient complaining about poor appetite and two patients complaining about acne. Another study analyzed the adverse reactions after DHEA intervention and found that patients developed acne (3.1%), darkened voice (1.9%), or hair loss (1.3%) after intervention. Therefore, the clinical application of androgen supplements (including DHEA and TTG) needs to be used under the guidance of clinical professionals, and appropriate doses need to be evaluated for different populations. Although we conducted subgroup analysis, due to the limited number of trials and small sample sizes in some subgroups, the data may not be sufficient to detect significant differences. In the future, exploratory studies should be performed on the degree of androgen priming in DOR patients after intervention with different androgen supplements. Moreover, it is deemed necessary to conduct more large-scale and comprehensive randomized controlled trials (RCTs) targeting the DOR population, in order to develop standardized plans for the selection and dosage determination of optimal androgen supplements.

## 6 Conclusions

The meta-analysis results indicated that intervention with DHEA or TTG prior to IVF to achieve androgen priming may have a positive effect on improving fertility in DOR women, with increasing the number of retrieved oocytes being the main benefit. The analysis results for RCTs indicated that androgen priming may improve the clinical pregnancy rate of DOR women, but no statistically significant improvement was shown. Although non randomized controlled trials had shown that androgen pretreatment may improve clinical pregnancy rates in DOR patients (*p*<0.05), such studies have a high risk of bias and low evidence certainty, and their results are only exploratory and of reference value. Overall, this meta-analysis provides exploratory implications for clinical decision-making, suggesting that androgen pretreatment (including DHEA and TTG) has potential beneficial trends for fertility in DOR patients. In the future, more high-quality, large sample, unified DOR diagnostic criteria and defined results from randomized controlled trials are needed to further validate current evidence.

## Data Availability

The original contributions presented in the study are included in the article/supplementary material. Further inquiries can be directed to the corresponding author.
